# Alterations of the Tongue in Diseases

**Published:** 1861-05

**Authors:** Carl Neidhardt


### SELECTED.

### ALTERATIONS OF THE TONGUE IN DISEASES.

BY DR. CARL NEIDHARDT.

Omitting other abnormal conditions of the tongue, Dr. Neidhardt arranges his remarks under three heads of coating, smoothness, and dryness.

*Abnormal Coatings* depend essentially upon a prolongation, loosening, and discoloration of the epithelial processes (*ciliœ, appendicœ epitheliœ*) of the papillæ. Whether a catarrhal condition is its cause, seems questionable; at least the places on the tongue free from coating show no hyperæmia. Certain it is that disordered nutrition occurs extraordinarily easy in the epithelium of the tongue. As to the *extent* of the coating, it is either total or partial; in the latter case it is either symmetrical, *i. e.*, covers corresponding portions of the tongue, or is unequal, sometimes entirely on one side. This, as well as the coating of the tongue in health, is owing to the motions of the tongue, and the friction it sustains. Thus the coating is found on only one-half of the tongue, as also in cases of one-side neuralgia trigemini. The symmetry of the coating often depends entirely on the form of the tongue, as f. i. in the conical; while unsymmetrical, partial coating, may be owing to accidental circumstances, as want of a tooth, projecting teeth, etc.

As variable as the extent, is also the amount or *thickness* of the coating. It also depends mechanically on the use and friction of the tongue, and is not proportionate to its roughness.

The *color* is at first grayish-white. It is changed from substances introduced from without, or from such as come from the body itself. Among the first, articles of food and medicines, as well as atmospherical dust, must be taken into consideration, besides special coloring matters. The discolorations from the body may arise from blood or biliary coloring matter. Blood extravasates on the mucous membrane of the tongue almost only in very severe diseases, when, in consequence of extreme dryness, fissures and rupture of superficial capillaries occur. The color may vary from reddish to black, constituting the rusty coating. The yellow coloration does not seem to be produced by biliary coloring matter, as is generally supposed, for it is found absent in the most intense icterus; it is owing, more likely, to the ingesta. The brownish color is connected with dryness of the tongue. The so-called *raspberry* tongue depends on the contrast in appearance of the filiform and fungiform papillæ. The latter having but little and translucent epithelium, are plainly to be seen as red points, when the former by the alteration of their epithelium produce the coating; the fungiform papillæ being especially numerous on the anterior half of the tongue, it is here, too, where the raspberry appearance is most observed. This appearance is by no means characteristic of scarlatina, as many believe; it is quite common in children, with slight, and even with normal coating, and has, therefore, neither in children nor in adults the slightest diagnostic value.

The *composition* of the abnormal coating resembles that of the normal coating of the tongue; *i. e.*, it consists of masses of epithelium, epithelial processes with granular cells that are thrown off, fat particles, fungi, and accidental mixtures.

The *cleaning of the tongue* usually proceeds gradually, the coating getting thinner and thinner; it is but seldom that the coating is thrown off in its whole thickness at once. Cleaning over the whole extent of the tongue never occurs at one time. The first clear portion is mostly the point of the tongue, where the cleaning proceeds to the edges and regularly backwards; sometimes the middle, very seldom the posterior portion, are first cleaned. The cause of this variation is, most likely, the varying forms of the frictional motion of the tongue; solution or absorption of the epithelial cells is an unsatisfactory explanation, and has never been proved; and a clearing up of the darkened or discolored cells is certainly very improbable.

The adherence of the coating to the tongue depends generally on its duration; reproduction after artificial removal is undesirable, as showing the continuance of the diseased condition.

The *taste* is either unchanged, especially with slight coating, or cannot be indicated by the words so often quoted.

The *diagnostic signification* of the coating is limited entirely to the fact indicating the existence of some diseased condition in general. [?] In a *prognostic* point of view, the disappearance of the coating is of value, as it is one of the first signs of the decrease of the disease. The significance of the dark coatings of a dry tongue will be considered under the latter heading.

b. *The Smooth or Shining Tongue.*—In consequence of the direction of the papillæ, the tongue, normally, is smooth on rubbing backwards, but rough to the touch, passing over it from the back, forwards. In disease, an extraordinary smoothness is not of unfrequent occurrence, accompanied, too, by reddening of the mucous membrane, and diminution of papillæ. The papillæ become smaller—and the smaller they become, the greater the reddening; for the epithelial covering becoming thinner at the same time, the blood is more easily seen through it. The abnormal smoothness of the tongue depends on the separation of the epithelial covering. Partial separation causes only partial smoothness. The smooth, shining tongue, being moist, reflects. Abnormal smoothness is found in acute as well as chronic diseases. In the former, it is without diagnostic significance, and, following generally the separation of the coating, has only the prognostic value of the cleaning of the tongue, as the epithelium is quickly reproduced in convalescence. In chronic diseases the smoothness indicates deficient reproduction of epithelium, from greatly disordered nutrition, without, however, indicating the nature of this disorder; it is unfavorable, prognostically, the disease being mostly incurable.

c. *The Dry Tongue.*—Dryness of the tongue is found in various degrees in health as well as in disease. It may be so excessive as to interfere with motion and with taste. The tongue is generally dry, partially, or over the whole extent, only on the back. If the smooth tongue becomes dry, it ceases to reflect. The color depends on the coating, which, as already mentioned, in extreme cases of dryness, becomes dark from fissures and rupture of the blood-vessels. For prognostic purposes, it must be noticed whether the dryness is only temporary or continues, and for how long it can be made to disappear by moistening. Temporary dryness is caused by breathing through the mouth, and is of no consequence. [Though the habit of some persons of breathing continually through the mouth is an unnatural and injurious one—the cause of snoring while sleeping, etc.—L. E.] When dryness lasts for days, and when it cannot be relieved by moistening the mouth, it is serious. Dryness usually commences at the middle, because that portion is least touched by the moisture of the mouth. Diminution of this moisture is the nearest cause of the dryness; the secretions of the mouth may be actually diminished, evaporation increased, or both these circumstances co-exist. Dryness is, therefore, almost always met with in unconscious patients, who do not feel its inconvenience. Its diagnostic importance is limited, excluding accidental drying circumstances, to the proof of some disease. Appearing in the course of acute disease, and not yielding but for the moment, to moistening, the prognoisis is rendered unfavorable; the greater the degree, the more unfavorable, of course. The becoming moist again of the tongue, especially when it continues, is a favorable symptom.

*Folds and Cracks of the Tongue.*—Folds on the tongue are of no importance whatever. They occur only after about the eighth year, generally, increase in number and depth with age, and are connected with the frequent change in the form and position of the tongue. If the tongue is coated, the papillæ in the folds remain coated longest, on account of their being less exposed to friction. Discolorations, for the same reason, are seen here longest. From the folds, cracks may follow a contraction of the mucous membrane in extreme dryness. As these cracks heal, cicatrices form, which are distinguished from the folds by the absence of papillæ.—*Am. Med. Monthly.*

